# Resistant *Rhodococcus* for Biodegradation of Diesel Fuel at High Concentration and Low Temperature

**DOI:** 10.3390/microorganisms12122605

**Published:** 2024-12-17

**Authors:** Irina Ivshina, Maria Kuyukina, Anastasiia Krivoruchko, Andrey Elkin, Tatyana Peshkur, Colin J. Cunningham

**Affiliations:** 1 Perm Federal Research Center, 13a Lenin Street, 614990 Perm, Russia; kuyukina@iegm.ru (M.K.); an220@mail.ru (A.E.); 2 Microbiology and Immunology Department, Perm State National Research University, 15 Bukirev Street, 614990 Perm, Russia; 3 Department of Civil and Environmental Engineering, University of Strathclyde, James Weir Building, Level 5, 75 Montrose Street, Glasgow G11XJ, UK; tanya.peshkur@strath.ac.uk (T.P.); colin.j.cunningham@strath.ac.uk (C.J.C.)

**Keywords:** diesel fuel, biodegradation, *Rhodococcus*, resistance, heavy contamination, low temperatures, biosurfactants, respirometry, growth kinetics, no catabolite repression

## Abstract

The resistance of 16 *Rhodococcus* strains to diesel fuel was studied. The minimal inhibitory concentrations of diesel fuel against *Rhodococcus* were 4.0–64.0 vol. % and 0.5–16.0 vol. % after 7 days of incubation in Luria–Bertani broth and a mineral “*Rhodococcus-*surfactant” medium, respectively. The three most resistant strains (*R. ruber* IEGM 231, IEGM 442 and *Rhodococcus* sp. IEGM 1276) capable of overcoming the toxicity of diesel fuel at a high (8.0 vol. %) concentration and at a low (4 °C) temperature were selected. Respiration activities, growth kinetics, and changes in the diesel fuel composition during the biodegradation process were elucidated using gas chromatography with mass spectrometry, respirometry, and Bradford analysis. Growth conditions were optimised for the improved biodegradation of diesel fuel by *Rhodococcus* cells using multifactor analysis. They included the simultaneous addition of 1.3 g·L^−1^ of granular sugar and 0.25 g·L^−1^ of yeast extract. The twofold stimulation of the biodegradation of individual hydrocarbons in diesel fuel (*n-*pentadecane, *n-*hexadecane and *n-*heptadecane) was demonstrated when glycolipid *Rhodococcus-*biosurfactants were added at a concentration of 1.4 g·L^−1^. A total removal of 71–91% of diesel fuel was achieved in this work.

## 1. Introduction

Diesel fuel is a widely used product of oil refinery. Accidental spills occur regularly during its production, transportation, storage, and exploitation, resulting in environmental contamination [1,2,3,4,5]. Although the dominant components of diesel fuel are medium and long chain alkanes [6,7,8], which are low or non-toxic and are considered to be the most available for microbial degradation [6,9,10], it remains one of the highly toxic petroleum products due to the presence of aromatic hydrocarbons and additives in its composition [11,12,13,14]. The contamination of soils with diesel fuel can lead to the inhibition of plant growth, reduction in soil respiration, increased mortality of soil invertebrates (e.g., nematodes), changes in the physical and mechanical properties of soils, and risks to human health [12,14,15,16,17,18].

Microorganisms play a key role in the degradation of petroleum hydrocarbons in the environment [13,19,20,21]. Actinomycetes of the genus *Rhodococcus* are among the most efficient biodegraders of hydrocarbons. They can utilise various petroleum components, including linear and branched C_2_–C_31_ alkanes, mono and polycyclic (both light and heavy) hydrocarbons and benzothiophenes, successfully degrade complex hydrocarbon mixtures (crude oil, gasoline, and diesel fuel), are tolerant to various stresses (low and high temperature and pH, high salinity, elevated concentrations of heavy metals and solvents, drought, starvation, and oxidative stress), and are easy to maintain [22,23,24,25,26,27].

The biodegradation of diesel fuel by *Rhodococcus* strains has been described in the literature. These bacteria have been shown to degrade 33% to 94% of diesel fuel in soil and water as monocultures or as part of consortia, to maintain high degradation activities at salinities up to 6% NaCl, and to have potential for diesel fuel desulphurisation [28,29,30,31,32,33]. However, the ability of *Rhodococcus* bacteria to degrade diesel fuel is not fully understood. Most biodegradation experiments with *Rhodococcus* cells have been carried out at low (0.1–2.0%) concentrations of diesel fuel and mesophilic (25–30 °C) temperatures [28,29,30,32,34]. The authors of [31] used diesel fuel concentrations of 3.0% and 4.0% and temperatures between 10 °C and 20 °C. Diesel fuel at concentrations < 3% has rather microbial promoting effects, stimulating the growth of heterotrophic prokaryotes, and leading to an increase in the metabolic activities of microorganisms. The negative effects of diesel fuel on microorganisms are more pronounced at higher concentrations [17]. At low temperatures, the toxicity of diesel fuel increased. This is related to the reduced evaporation of toxic volatile components, increased viscosity of diesel fuel, and prevented photovolatilisation of hydrocarbons due to the high surface albedo from snow cover [35]. As a result, hydrocarbons persist in the environment for long periods of time, particularly in cold climates such as the Arctic, Siberia, or Antarctica [14,17,35,36,37]. Therefore, the discovery of new strains of *Rhodococcus* capable of the biodegradation of diesel under such conditions may be useful for bioremediation.

The effect of nitrogen sources on this process has been carefully assessed [31]. A less well understood effect is the influence of additional carbon sources on the biodegradation of diesel fuel. Supplementation with NH_4_Cl, urea, poultry manure, or soluble organic matter facilitated 1.5–4.0 times the removal of diesel fuel [29,31,35,36,37,38]. It has been shown that more available carbon sources (yeast extract and glucose) accelerated the bioconversion of less available hydrophobic substrates ((–)-isopulegol, diclofenac, and drotaverine hydrochloride) by *Rhodococcus* cells, although these substrates could be used as the sole carbon sources [39,40,41].

The effects of externally added biosurfactants (the secondary metabolites, which are produced by bacteria in the presence of hydrophobic compounds to facilitate their utilisation) on diesel fuel biodegradation by *Rhodococcus* has not been studied. However, other diesel-fuel-degrading bacteria have been exposed to biosurfactants and the influence of these biomolecules on their degradation capabilities has been demonstrated. In particular, the supplementation of *Achromobacter* sp. 4(2010) and *Rahnella* sp. EK12 cells with rhamnolipids and saponins resulted in an up to twofold increase in diesel fuel biodegradation [42,43]. Positive, neutral, and negative effects of externally added rhamnolipids have been shown on the biodegradation of diesel fuel and its blends with biodiesel by a bacterial consortium, depending on the blend composition [44].

The aims of this study were as follows: to evaluate the resistance characteristics of *Rhodococcus* actinomycetes to diesel fuel; to study the biodegradation activities of *Rhodococcus* towards diesel fuel as the sole growth substrate and in the presence of additional carbon sources (specifically glucose and yeast extract); to identify new, promising biodegraders of diesel fuel, active at high (≥3%) concentrations of this pollutant and at a low (4 °C) temperature; and to assess externally added biosurfactants (glycolipids produced by *Rhodococcus* cells) in facilitating the diesel fuel biodegradation by these bacteria.

## 2. Materials and Methods

### 2.1. Chemicals

Mineral salts, solvents, Luria–Bertani broth (LB), LB agar (LBA), iodonitrotetrazolium violet (INT), hydrocarbons (*n-*alkanes), NaOH, and the Bradford reagent were >97% pure and were purchased from Sigma-Aldrich, Inc. (St. Louis, MO, USA). Winter diesel fuel ECTO grade (Lukoil-Permnefteorgsintez, Perm, Russia), granular sugar (Agrocompany VESNA LLC, Sergach, Russia), and yeast extract (Microgen, Moscow, Russia) were used as carbon sources. Crude glycolipid *Rhodococcus-*biosurfactants were obtained from *Rhodococcus ruber* IEGM 231 cells grown in the presence of 3 vol. % of *n-*hexadecane as previously described [45,46]. For this, the upper hydrophobic layer of a bacterial culture was treated with ultrasound at 44 kHz, 0.7 A, and 4 °C for 30 min. The glycolipids were then extracted using methyl tertiary-butyl ether (MTBE).

### 2.2. Bacterial Strains

A total of 18 strains of *Rhodococcus,* isolated from different clean and polluted environments and maintained in the Regional Specialised Collection of Alkanotrophic Microorganisms (acronym IEGM, WDCM number 768, http://www.iegmcol.ru/, accessed on 15 November 2024) were used in this study (Table 1). The IEGM collection maintains more than 3000 pure, identified, non-pathogenic, and well-described hydrocarbon-oxidising actinomycetes, and this is a valuable resource for the targeted selection of strains based on their isolation sites, tolerances, and desired functional activities, followed by further biodegradation experiments [25,27,47]. *Rhodococcus* cells, pre-grown on LBA and then suspended in 0.5% (*w*/*w*) NaCl at a concentration of 1×10^8^ colony forming units (CFU)·mL^−1^ were used as inocula.

### 2.3. Toxicity Tests

Toxicity tests were performed in polystyrene 96-well microplates (Medpolymer, St. Petersburg, Russia) with the LB or a mineral “*Rhodococcus-*surfactant” medium (RS) supplemented with diesel fuel at concentrations of 0.25, 0.5, 1.0, 2.0, 4.0, 8.0, 16.0, 32.0, or 64.0 vol. %. The composition of the RS was as follows (http://www.iegmcol.ru/medium/med11.html, accessed 15 November 2024): KH_2_PO_4_—2.0 g·L^−1^, K_2_HPO_4_—2.0 g·L^−1^, KNO_3_—1.0 g·L^−1^, (NH_4_)_2_SO_4_—2.0 g·L^−1^, NaCl—1.0 g·L^−1^, MgSO_4_·7H_2_O—0.2 g·L^−1^, CaCl_2_—0.02 g·L^−1^, yeast extract—1.00 g·L^−1^, and the trace element solution [FeCl_3_·7H_2_O—1.5 g·L^−1^,H_3_BO_3_—0.1 g·L^−1^, ZnSO_4_·7H_2_O—0.01 g·L^−1^, Co(NO_3_)_2_·6H_2_O—0.05 g·L^−1^, CuSO_4_·5H_2_O—0.005 g·L^−1^, MnCl_2_·4H_2_O—0.005 g·L^−1^]—1 mL·L^−1^, pH 6.8–7.0. A 15 μL cell suspension with a concentration of 1×10^8^ CFU·mL^−1^ was added to the microplates. The total volume of liquid in the wells was 150 μL, and the initial cell concentration was 1×10^7^ CFU·mL^−1^. The inoculated microplates were incubated in a Titramax 1000 incubator (Heidolph Instruments, Schwabach, Germany) at 600 min^−1^ and 28 °C for 3 or 7 days. Cell viability was estimated by staining with INT. For this, 45 μL of 0.2% (*w*/*w*) INT solution in water was added to the microplates. INT is a strong oxidiser. It competes with molecular oxygen in respiratory chains. INT reduction produces insoluble red-violet formazan. The appearance of a red-violet colour after 2 h of staining indicated the presence of viable, respirating cells [48]. The lowest concentration at which no red-violet colour appeared after 3 days of incubation was the minimal inhibitory concentration (MIC). If no colour appeared after 7 days, this concentration was considered bactericidal. Inoculated LB and RS supplemented with 3 vol. % of *n-*hexadecane were biotic controls. Uninoculated LB and RS supplemented with 0.5 vol. % of diesel fuel were abiotic controls.

### 2.4. Biodegradation of Diesel Fuel and Other Growth Conditions

Biodegradation experiments were performed in 100 mL Erlenmeyer flasks with 20 mL of the RS medium supplemented with 0.5, 1.0, 2.0, or 8.0 vol. % diesel fuel at 160 rpm and 4 °C or 28 °C for 3–8 days. Inocula were added at 1 vol. % and the initial cell concentration was 1×10^6^ CFU·mL^−1^. The visual monitoring of the cultures was performed during and at the end of biodegradation using an Axiostar Plus microscope (Carl Zeiss, Oberkochen, Germany) at ×1000 magnification to verify microbiological purity. Uninoculated flasks containing diesel fuel were abiotic controls. In some variants, *Rhodococcus-*biosurfactants were added to all trials at a concentration of 1.4 g·L^−1^, corresponding to 2× critical micelle concentrations (CMC) [46]. To estimate the effects of other carbon sources on the biodegradation process, *Rhodococcus* cells were cultured simultaneously in the presence of diesel fuel, sugar, and yeast extract. Sugar concentrations were 1.3, 2.5, 5.0, and 10.0 g·L^−1^. Yeast extract concentrations were 0.25, 0.50, and 1.00 g·L^−1^. For relevant comparisons, cells were also cultured in the simultaneous presence of two substrates. The number of cells after biodegradation was determined by plating the cultures on LBA, incubating at 28 °C for 2 days, and calculating as CFU·mL^−1^.

### 2.5. Analysis of Diesel Fuel

Residual diesel fuel was extracted from cultures with chloroform. The sample (20 mL) and solvent (20 mL) were thoroughly mixed for 20–30 s and then allowed to stand for 2 min. The bottom phase was collected, the extraction was repeated 2 more times, and the chloroform fractions were combined. Water was removed from the extracts with anhydrous Na_2_SO_4_, and extracts were filtered and processed for solvent evaporation in pre-weighed Soxhlet glasses using a Büchi Extraction System B-811 (Buchi, Flawil, Switzerland). The glasses were allowed to weather in a fume cupboard for 24 h and weighed again. The difference between the weights of glasses before and after extraction was the weight of the residual fuel.

For GC-MS, residual diesel fuel samples were dissolved in 10 mL of chloroform and the analysis was performed using an Agilent 6890N gas chromatograph (Agilent Technologies, Santa Clara, CA, USA) equipped with a 30 m HP-5MS column with an internal diameter of 0.25 mm, a film thickness of 0.25 μm, and an Agilent MSD 5973N quadrupole mass spectrometer (Agilent Technologies, Santa Clara, CA, USA). The 1 μL sample was injected into the injection port, which was held at 250 °C. The initial oven temperature was 80 °C and was held for 2 min; then, the oven was heated to 300 °C at 10 °C·min^−1^. The helium flow was 1 mL·min^−1^. The mass spectrometer was operated in electron ionisation mode in the *m*/*z* range of 40–600 and data acquisition were performed in selective ion mode. The acquisition rate was 1.36 scans per second with a detector voltage of 1200 V. Hydrocarbon identification and quantification were based on ion fragmentation and retention times compared to the model mixture of *n-*alkanes C_10_–C_19_ at equimolar concentrations. Calibration curves between peak squares and concentrations of individual *n-*alkanes in the mixture were constructed and used to calculate concentrations of these hydrocarbons in the residual diesel fuel. Only data from peaks of quality ≥90 were extracted for the calculations.

### 2.6. Determination of Glucose

The concentration of glucose was determined using a “Photoglucose”glucose oxidase kit (LLC “Impact”, Moscow, Russia). The working solution containing glucose oxidase, peroxidase, and 4-aminoantipyrine was prepared according to the manufacturer’s instructions. In addition, 2 mL of the working solution was thoroughly mixed with 25 μL of cell-free culture medium and incubated at room temperature for 25 min. The absorbance at 500 nm was then measured using a Lambda EZ201 spectrophotometer (Perkin Elmer, Shelton, CO, USA) in comparison with calibration and control samples prepared in the same way. The calibration sample contained 2 mL of the working solution and 25 μL of the calibrator (10 mmol·L^−1^ glucose in 0.15% benzoic acid). The control sample contained 2 mL of the working solution and 25 μL of distilled water. The concentration of glucose was calculated using the following formula:

(1) $$C=\frac{E_{0}}{E_{x}}\times 10,$$

where *C*—concentration of glucose, mmol·L^−1^; *E*_0_—A_500 nm_ of the experimental sample (culture medium); *E_x_*—A_500 nm_ of the calibration sample; and 10—concentration of glucose in the calibrator, mmol·L^−1^.

### 2.7. Bradford Analysis

**Figure 1:**
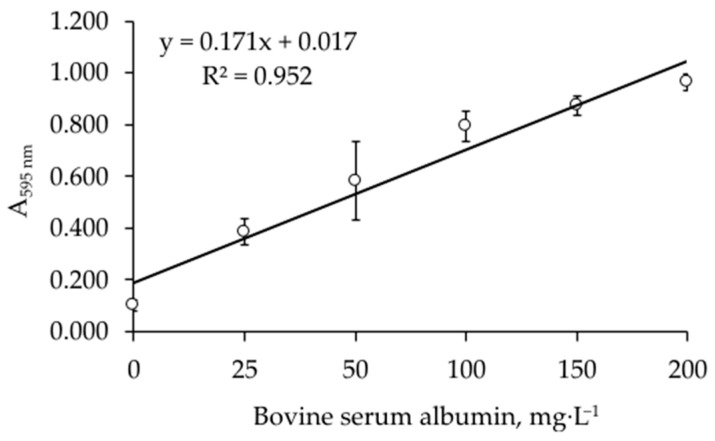
Calibration curve between concentration of bovine serum albumin and A_595 nm_ for Bradford analysis.

Total protein was measured daily in growing cultures during the biodegradation of diesel fuel. First, diesel fuel was washed out of the cells. For this, 20 mL of isopropanol was added to 20 mL of culture, the mixture was centrifuged at 3000× *g* rpm for 10 min, and the supernatant was discarded. The 100 μL of 0.05 M NaOH was added to the cell pellet, and 3 cycles of heating in a water bath at 96 °C for 15 min and freezing at –20 °C for 15 min were performed to disrupt the cells. Then, 1 mL of milliQ water was added, and the 50 μL suspension obtained was mixed with the 500 μL Bradford reagent. The mixture was incubated at room temperature for 10 min, the absorbance at 595 nm was measured (if necessary, the sample was diluted), and the protein concentration was calculated using a calibration curve (Figure 1). The abiotic control (RS with diesel fuel, without cells), which was treated in the same way, was used as a blank sample.

Kinetic growth parameters were calculated using the following formulae:

(2) $$Y=\frac{X}{S},$$

(3) $$X=X_{max}-X_{0},$$

(4) $$\mu =\frac{X_{max}}{t\times X_{0}}$$

where *Y*—economic coefficient; *X*—biomass yield, g proteins·L^−1^; *S*—amounts of substrate consumed, g·L^−1^; *X_max_*—maximum amounts of proteins, g·L^−1^; *X*_0_—initial amounts of proteins, g·L^−1^; *μ*—specific growth rate, h^−1^; and *t*—exponential phase time interval, h.

### 2.8. Respirometry

The dynamics of *Rhodococcus* cell respiratory activity during diesel fuel biodegradation was monitored using a Micro-Oxymax 10-channel respirometer equipped with O_2_ and CO_2_ sensors (Columbus Instruments, Columbus, OH, USA). The analysis was performed in 250 mL bottles containing 100 mL RS supplemented with 1.0, 2.0, or 3.0 vol. % diesel fuel under magnetic stirring (300 rpm) at 28 °C for 3 days. The initial cell concentration was 1·10^6^ CFU·mL^−1^. Non-inoculated bottles were used as abiotic controls. Rates of O_2_ consumption and CO_2_ production were calculated as measures of respiratory activity.

### 2.9. Statistics and Multifactor Analysis

All experiments were performed in 3–8 replicates. Statistica version 13.5.0.17 (TIBCO Software Inc., Palo Alto, CA, USA) was used to calculate basic statistics and perform multifactor analysis. Multifactor analysis was used to estimate the effects of carbon sources on the biodegradation of diesel fuel by *Rhodococcus* cells. The conditions were standardized as follows: the central and two boundary parameters were designated as levels 0, +1, and −1, respectively (Table 2); and all combinations of carbon source concentrations (27 in total) were indexed (Table S1). Pareto diagrams were constructed to show the apparent effects and their statistical significance.

## 3. Results

### 3.1. Toxicity of Diesel Fuel

The results of the toxicity studies are presented in Table 3. The inhibitory concentrations of diesel fuel in the LB (MICs) for the *Rhodococcus* strains tested varied in a wide range from 0.5 to 64.0 vol. %. For 50% of the strains, these concentrations were bactericidal, and no growth was detected after 7 days of exposure. Meanwhile, the other 50% of the *Rhodococcus* strains grew in LB supplemented with diesel fuel at a concentration equal to the MIC after 7 days. For these strains, diesel fuel had a bacteriostatic effect. This effect was particularly typical for strains with MICs ≤ 4.0 vol. %. After 7 days, the inhibitory concentrations increased by eight to sixty-four-fold; this result was probably related to the adaptation of the cells and their subsequent growth (Table 3). Nevertheless, based on the MIC values, *R. rhodochrous* IEGM 639, *R. ruber* IEGM 231, IEGM 442, IEGM 1263, and *Rhodococcus* sp. IEGM 1276 were the strains most resistant to diesel fuel (MICs = 32.0–64.0 vol. %). *R. jostii* IEGM 60 and *R. ruber* IEGM 234 were the strains most susceptible to diesel fuel (MIC = 0.5 vol. %) (Table 3).

The toxicity of diesel fuel was mainly higher in a mineral RS medium compared to the LB. In the RS medium, where diesel fuel was the sole carbon source, most *Rhodococcus* strains were two to sixty-four times less resistant to its action than in LB (Table 3). The inhibitory concentrations of diesel fuel in the RS medium were no higher than 16.0 vol. %, and a bactericidal effect of diesel fuel was demonstrated against all strains. Two strains, *R. erythropolis* IEGM 251 and *R. ruber* IEGM 234, were exceptions. The toxicity of diesel fuel against *R. erythropolis* IEGM 251 was the same in both media, and *R. ruber* IEGM 234 was twice as resistant in the RS medium than in the LB (Table 3). *R. ruber* IEGM 442 was the most resistant strain, with inhibitory concentrations of diesel fuel of 32.0 and 16.0 vol. % in the LB and RS medium, respectively.

### 3.2. Biodegradation of Diesel Fuel by Resistant Rhodococcus Strains

For the biodegradation experiments, three strains consisting of *R. ruber* IEGM 231, IEGM 442, and *Rhodococcus* sp. IEGM 1276 were selected based on their resistance to diesel fuel. The growth of all three strains in the presence of diesel fuel was detected at both 4 °C and 28 °C. All three were able to use diesel fuel as a growth substrate at a concentration of 2 vol. %. However, the percentage of diesel fuel degradation depended on the temperature (Table 4). The highest (59%) percentage of the biodegradation after 8 days was found for *Rhodococcus* sp. IEGM 1276 at 4 °C. This strain was apparently psychrophilic, since its degradation activity towards diesel fuel at 28 °C was two times lower than at 4 °C and was only 33% (Table 4). In contrast, *R. ruber* strains were mesophilic. Their oxidation activities towards diesel fuel were three to four times lower at 4 °C than at 28 °C, corresponding to 10–15% and 42–44%, respectively (Table 4).

As seen from Table 4, microbial degradation was not the only contributor to the total removal of diesel fuel. Significant amounts of diesel fuel were lost abiotically due to the evaporation of volatile components such as alkanes with a chain length of ≤10 carbon atoms, monoaromatic hydrocarbons, and naphthalene [11]. Abiotic losses were three times lower at 4 °C than at 28 °C, at 14% and 42–43%, respectively (Table 4). At 28 °C, evaporation was important, and the total removal of diesel fuel was 75–86% (Table 4). At 4 °C, the participation of an actively degrading strain such as *Rhodococcus* sp. IEGM 1276 was more important than evaporation, and the total removal of diesel fuel was similar (73%). In contrast, the total removal of diesel fuel at 4 °C in the presence of mesophilic strains (*R. ruber* IEGM 231 and IEGM 442) was only 28–29%.

**Figure 2:**
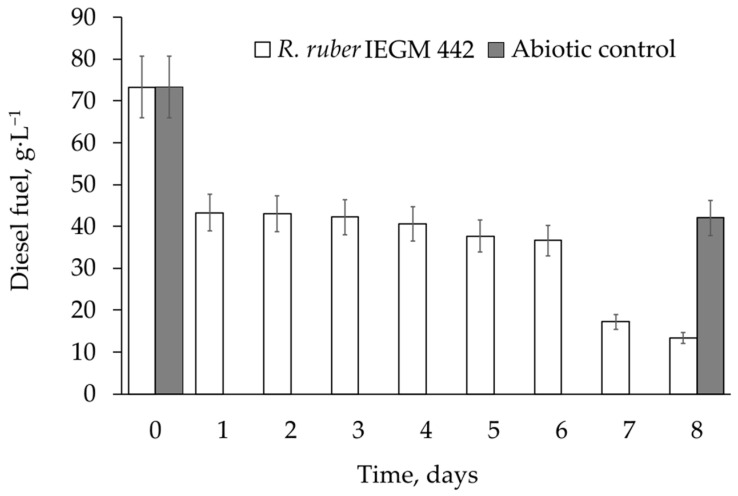
Dynamics of total removal of diesel fuel at a concentration of 8.0 vol. % and temperature of 28 °C in the presence of *R. ruber* IEGM 442 cells.

The most resistant strain, *R. ruber* IEGM 442, efficiently degraded diesel fuel at a high concentration (8.0 vol. %), while maintaining the same oxidising activity as at 2.0 vol. %. The percentages of diesel fuel biodegradation by *R. ruber* IEGM 442 cells were 42% and 40% at concentrations of 2.0 vol. % and 8.0 vol. %, respectively (Table 4). At the maximum (8.0 vol. %) concentration, the biodegradation process showed three distinct phases. A significant decrease in the diesel fuel concentration to 41% was observed after 1 day of degradation. Apparently, this was related to the abiotic losses of the fuel, which reached their maximum after 1 day. For the next 5 days, the concentration of diesel fuel did not change, and it was probably the adaptation phase for the cells. On the 7th–8th day, the removal of diesel fuel was intensified, apparently due to degradation by the adapted *R. ruber* IEGM 442 cells (Figure 2).

### 3.3. GC-MS Analysis of Residual Diesel Fuel and Influence of Rhodococcus-Biosurfactants

**Figure 3:**
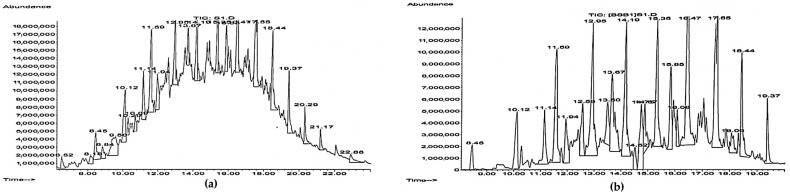
GC-MS chromatograms of original (**a**) and residual diesel fuel after 8 days of biodegradation using *R. ruber* IEGM 442 cells at 28 °C (**b**).

As shown by GC-MS analysis, the residual diesel fuel consisted mainly of alkanes C_9_–C_19_ (Figure 3). Unseparated peaks on the chromatograms were evidence of large amounts of different hydrocarbon isomers, which are typical for diesel fuels [6,7,8,11]. However, linear alkanes predominated among the hydrocarbons detected, as they were represented by the highest peaks (Figure 3). The lightest and most volatile components (retention time < 8.45 min) disappeared in the residual fuel and, apparently due to their volatilisation and partial degradation, the residual diesel fuel was better separated than the original product (Figure 3).

**Figure 4:**
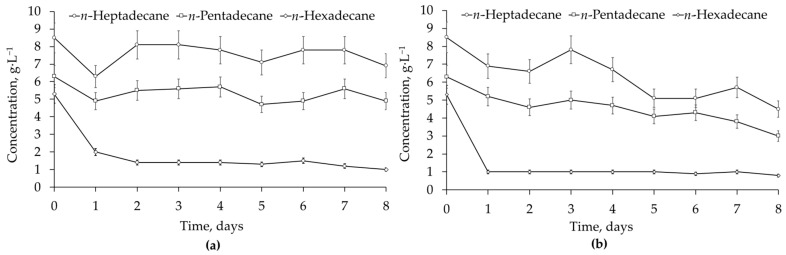
Biodegradation of individual hydrocarbons in diesel fuel at its concentration of 8.0 vol. %, at 28 °C in 8 days by *R. ruber* IEGM 442 cells without (**a**) and in the presence (**b**) of *Rhodococcus-*biosurfactants added at a concentration of 1.4 g·L^−1^.

Three *n-*alkanes, *n-*pentadecane (retention time 14.19 min), *n-*hexadecane (retention time 15.35 min), and *n-*heptadecane (retention time 16.47 min), were detected in all residual diesel fuel samples. By analysing the dynamics of the concentration changes of these representative *n-*alkanes, the influence of *Rhodococcus-*biosurfactants was revealed. Supplementation with *Rhodococcus-*biosurfactants facilitated the biodegradation of *n-*alkanes, and this effect was particularly evident at high diesel fuel concentrations. Without biosurfactants, the concentrations of *n-*pentadecane and *n-*heptadecane did not change significantly after 8 days of biodegradation of 8.0 vol. % diesel fuel by the *R. ruber* IEGM 442 cells. Their degradation under the same conditions but in the presence of biosurfactants was 52% and 47%, respectively (Figure 4). The biodegradation of *n-*hexadecane differed by 7% between the experiments and was 79% and 86% without and in the presence of *Rhodococcus-*biosurfactants, respectively. Furthermore, *n-*hexadecane was removed more rapidly in the presence of *Rhodococcus-*biosurfactants, with the process completed in 1 day. Without biosurfactants, no further removal of *n-*hexadecane was registered after 2 days (Figure 4).

### 3.4. Respiration and Growth Kinetics of Rhodococcus Cells at Biodegradation of Diesel Fuel

**Figure 5:**
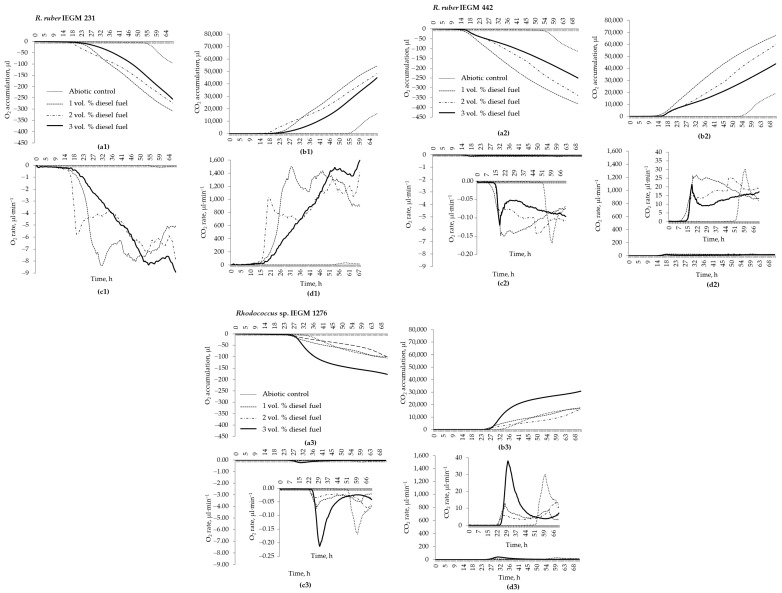
Respiratory activities of *Rhodococcus* strains during the biodegradation of different concentrations of diesel fuel at 28 °C. The parameters measured were as follows: (**a**) consumption of O_2_, μL; (**b**) production of CO_2_, μL; (**c**) rate of O_2_ consumption, μL·min^−1^; and (**d**) rate of CO_2_ production, μL·min^−1^.

In order to better assess the effects of the diesel fuel concentration on the biodegradation process, the respiratory activities of *Rhodococcus* cells were analysed. Concentrations of 1.0, 2.0, and 3.0 vol. % diesel fuel were used in these experiments. In terms of respiratory activities, the biodegradation of diesel fuel by *Rhodococcus* cells could be divided into three phases: a lag phase with barely detectable respiration, a short phase of intense and dramatically increasing respiratory activity, and a relatively stable phase with one or more peaks of respiratory activity (Figure 5). The concentration of diesel fuel did not significantly affect the duration of a lag phase (it rather depended on the strain specificity), but it did affect the respiratory rates, O_2_ consumption/CO_2_ production, the duration of the second phase, and the dynamics (e.g., the number and the time of appearance of the respiratory activity peaks) of the third phase (Figure 5).

Among the strains studied, *R. ruber* IEGM 231 had the highest respiratory rates (up to 8.923 μL O_2_·min^−1^ and 1600 μL CO_2_·min^−1^). The maximum values of the rates depended on the diesel fuel concentration and were reached at different times: 17 h, 30 h, and 50 h at 1.0 vol. %, 2.0 vol. %, and 3.0 vol. % diesel fuel, respectively (Figure 5). Two other strains, *R. ruber* IEGM 442 and *Rhodococcus* sp. IEGM 1276, were 42–115 times less active than *R. ruber* IEGM 231. The maximum rates of O_2_ consumption and CO_2_ production for these strains were only 0.074–0.150 μL·min^−1^ and 13–38 μL·min^−1^, respectively (Figure 5).

However, the greatest changes in O_2_ and CO_2_ were observed at the biodegradation of 1.0 vol. % (381 μL O_2_ and 67,581 μL CO_2_) and 2.0 vol. % (339 μL O_2_ and 59,248 μL CO_2_) of diesel fuel by the *R. ruber* IEGM 442 cells. This strain also exhibited the shortest lag phase, which did not exceed 13 h, whereas the lag phases of *R. ruber* IEGM 231 and *Rhodococcus* sp. IEGM 1276 were 13–17 h and 22–24 h, respectively (Figure 5).

The lowest changes in respiratory gases (99–105 μL O_2_ and 16,516–17,597 μL CO_2_) were observed during the biodegradation of diesel fuel by the *Rhodococcus* sp. strain IEGM 1276. Interestingly, the amounts of O_2_ consumed and CO_2_ released, as well as the respiratory rates, were two and three times higher for this strain at the biodegradation of 3.0 vol. % diesel fuel than at its lower concentrations (Figure 5). The dependence of respiratory activities on diesel fuel concentration was more typical for the *R. ruber* IEGM 231 and IEGM 442 cells. The O_2_ consumption and the CO_2_ emission in the presence of these strains decreased proportionally with increasing diesel fuel concentrations. The consumption of O_2_ and production of CO_2_ by *R. ruber* IEGM 442 at 3 vol. % diesel fuel and IEGM 231 at all three diesel fuel concentrations were similar and varied within narrow ranges of 250–307 μL and 43,785–54,351 μL, respectively (Figure 5).

**Figure 6:**
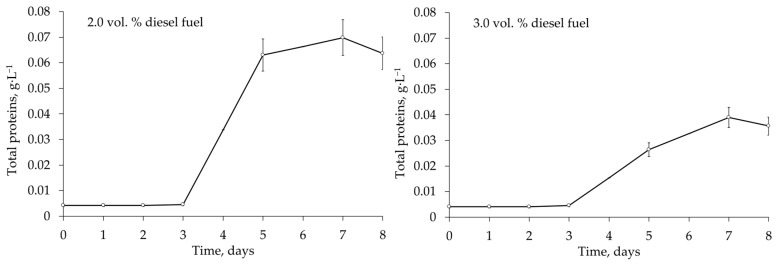
Growth kinetics of *Rhodococcus* sp. IEGM 1276 cells in the presence of 2.0 and 3.0 vol. % diesel fuel at 4 °C.

At 4 °C, the kinetics of the growth of *Rhodococcus* sp. IEGM 1276 in the presence of diesel fuel was determined by measuring the total amount of protein. The observed changes in the protein concentration corresponded to the growth phases and confirmed that the concentration of diesel fuel at 3 vol. % was too high and did not favour the growth of *Rhodococcus* sp. IEGM 1276 (Figure 6), in contrast to the respirometry data (Figure 5). Growth kinetic parameters for *Rhodococcus* sp. IEGM 1276 cells at 3 vol. % diesel fuel were as follows: economic coefficient (showing the conversion rate of substrate to protein) of 0.005, specific growth rate of 0.312 h^−1^, and yield of 0.039 g·L^−1^. At 2 vol. % diesel fuel, the economic coefficient was twelve times higher, the specific growth rate was two times lower, and the yield was two times higher than at 3 vol. % with corresponding values of 0.069, 0.138 h^−1^, and 0.070 g·L^−1^ (Table 5).

### 3.5. Influence of Additional Carbon Sources on Biodegradation of Diesel Fuel by Rhodococcus Cells

Rhodococci metabolised carbohydrates in the form of granular sugar in the presence of diesel fuel (Table 6). In addition, the simultaneous presence of sugar and diesel fuel stimulated the growth of *Rhodococcus* cells. The number of cells after 7 days of cultivation in the presence of sugar or diesel fuel as sole growth substrates was 0.12·10^7^ CFU·mL^−1^ and 1.65·10^7^ CFU·mL^−1^, respectively. The cell number increased 1.5–20.0 times when both substrates were used simultaneously (Table 7). Since the percentage of sugar removal was almost the same in the presence and absence of diesel fuel, this was evidence that both substrates were used simultaneously, and both contributed to the growth and cell number of *Rhodococcus* cells. The effect of the yeast extract was initially unclear and a further multifactor analysis was performed to better estimate the effect of each carbon source on *Rhodococcus* cell growth.

**Figure 7:**
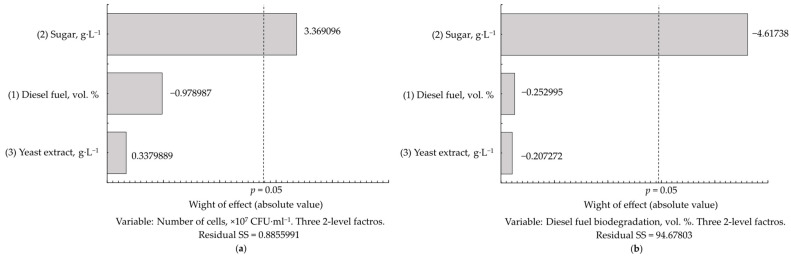
Pareto diagrams showing the effects of sugar, diesel fuel, and yeast extract concentrations on the number of *R. erythropolis* IEGM 587 cells (**a**) and the biodegradation of diesel fuel (**b**).

As seen from the Pareto diagrams, the sugar concentration had a strong effect on the number of *Rhodococcus* cells and the biodegradation of diesel fuel. Sugar stimulated the cell growth but interfered with diesel fuel degradation, and its effect on diesel fuel biodegradation was greater than on growth (Figure 7). The highest (3.15·10^7^–4.60·10^7^ CFU·mL^−1^) cell numbers were obtained at a sugar concentration of 5.0 g·L^−1^. And the lowest (16–65%) percentages of diesel fuel removal were also obtained at this sugar concentration (Table 8). Sugar at concentrations > 2.5 g·L^−1^ inhibited the biodegradation process by 9–60%. Diesel fuel at concentrations of 0.5–2.0 vol. % and yeast extract had minor, statistically insignificant effects on growth and biodegradation efficiency (Figure 7, Table 8). The highest (91%) percentage of diesel fuel biodegradation was achieved at sugar, yeast extract, and diesel fuel concentrations of 2.5 g·L^−1^, 1.00 g·L^−1^, and 0.5 vol. %, respectively. Similar biodegradation efficiencies of 88% were obtained when sugar and yeast concentrations were two to four times lower (to reduce economic costs) and the diesel fuel concentration was 2.0 vol. % (Table 8). Optimal growth conditions were 1.3–2.5 g·L^−1^ of granular sugar and 0.25 g·L^−1^ (the minimum value) of yeast extract, which allowed the biodegradation of 57–91% of diesel fuel at its concentration of 0.5–2.0 vol. %. The preferred sugar concentration was 1.3 g·L^−1^. The biodegradation of diesel fuel at this concentration was constantly high, varying only between 71% and 88%.

## 4. Discussion

The toxicity of diesel fuel to 16 *Rhodococcus* strains was determined in this study. Two strains, *R. erythropolis* IEGM 275 and 587, were not included in this analysis because they were already used by our team as degraders of diesel fuel and were known to grow at 2 vol. % of this petroleum product (unpublished data). No species dependency of the resistance of the *Rhodococcus* strains studied was observed, making predictions of strain resistance based on biological characteristics of species inapplicable. For example, the most sensitive (IEGM 234, MIC = 0.5 vol. % diesel fuel) and the most resistant (IEGM 231, IEGM 442 and IEGM 1263, MICs = 32.0–64.0 vol. % diesel fuel) strains were found among the representatives of the *R. ruber* species with a 128-fold difference between the least and the highest toxic concentrations. Extreme levels of resistance were also found in other species (see Table 3). However, for sensitive strains, MICs were typically bacteriostatic concentrations and the inhibitory effects of diesel fuel in 3 days should be considered as underestimated. More relevant toxic effects of diesel fuel towards *Rhodococcus* cells were registered after 7 days. Comparing the resistance of strains to diesel fuel in the LB after one week, no species specificity was revealed again, but the differences between sensitive and resistant strains were less contrasting. They were no more than eight-fold and looked more like normal variations. Only *R. ruber* strain IEGM 234 was 32 times more sensitive to diesel fuel than strain IEGM 231 (see Table 3), which could be related to the biological specificity of *R. ruber* IEGM 234. The theoretical bases for the differences in strain resistance could be the different permeability of the cell wall due to its hydrophobicity or thickness, different time to respond to the toxicant, and specificity of regulatory mechanisms.

It is common practice to determine the toxicity of chemical compounds in rich culture media containing sufficient amounts of nutrients and available growth substrates (e.g., LB, nutrient broth and tryptic soy broth). The inhibition of growth in these media is related to the action of a toxicant and not to a lack of nutrients/elements, a long lag phase, auxotrophic conditions, cell efforts to utilise a difficult substrate, starvation, or any other stress. Consequently, the resistance of *Rhodococcus* cells to diesel fuel in the mineral RS medium, where diesel fuel was both a toxicant and a growth substrate, was predictably lower than in the LB, and all inhibitory concentrations were bactericidal. Six out of sixteen strains (*R. erythropolis* IEGM 1189, *R. jostii* IEGM 60, *R. opacus* IEGM 717, IEGM 1157, *R. qingshegii* IEGM 1359, and *R. rhodochrous* IEGM 1138) almost completely lost their tolerance to diesel fuel in the RS medium, with MIC values of only 0.5–1.0 vol. % (Table 3). Compared to other species in the mineral medium, *R. ruber* seemed to be the most resistant to diesel fuel and was inhibited by no less than 8.0 vol. % diesel fuel (see Table 3). This was in agreement with our previous works. *R. ruber* was more resistant to monoaromatic hydrocarbons and survived long storage (lyophilisation and cryopreservation) better than other *Rhodococcus* species [25,27]. This species is known to produce carotenoid pigments, and its strains have a bright orange colour. Carotenoids have been reported to protect *Rhodococcus* cells from UV irradiation, cold, heat, and oxidative stress, and to be involved in biofilm development [23,27]. We suggest that pigments may be involved in the protection of *R. ruber* cells from diesel fuel.

The resistance of most *Rhodococus* strains to diesel fuel was impressively high and they survived in culture media that could be one third of the hydrophobic toxic substance. Moreover, the cells continued to grow; at least, the red-violet colour in wells with non-inhibitory concentrations of diesel fuel was as bright as that in biotic controls (Figure S1). Increased tolerance to toxic hydrophobic compounds is typical for *Rhodococcus*. Rhodococci survive in the presence of 20–80 vol. % polar (ethanol and butan-1-ol) and non-polar (toluene, *n-*hexane and *n-*decane) solvents. Their ability to resist the toxic effects of solvents is associated with an increase in the relative surface area, a decrease in the cell wall rigidity upon the contact with solvents, and the involvement of efflux pumps [49].

Three strains were selected for biodegradation experiments: *R. ruber* IEGM 231 and IEGM 442, which showed a high (inhibition concentrations were 8.0–64.0 vol. %) resistance to diesel fuel in both the LB and RS medium, and *Rhodococcus* sp. IEGM 1276, which was not very resistant to diesel fuel in the RS medium but showed the highest (MIC = 64.0 vol. %) resistance in the LB (see Table 3). These strains degraded from 33% to 44% of 2.0 vol. % diesel fuel at 28°C in 8 days (Table 4). Combined with the removal of diesel fuel due to evaporation (abiotic losses), the total removal of this petroleum toxicant was between 75% and 86% (see Table 4), which was similar to published efficiencies [6,29,30,31,32,33]. Particularly promising results for ecobiotechnology were obtained with *R. ruber* IEGM 442, which was able to degrade 8.0 vol. % diesel fuel with the same efficiency as at 2.0 vol. %, and *Rhodococcus* sp. IEGM 1276, which was able to degrade 59% of 2.0 vol. % diesel fuel at 4 °C. Notably, the last strain was psychrophilic and was two times less active at 28 °C (Table 4). Thus, both strains were promising for bioremediation: *R. ruber* IEGM 442—at a mesophilic temperature (28 °C) but with high diesel fuel contamination (up to 8 vol. %), and *Rhodococcus* sp. IEGM 1276—with lower contamination levels (up to 2–3 vol. %) but under cold (4 °C) conditions. Although the respiratory activity of *Rhodococcus* sp. IEGM 1276 increased with increasing diesel fuel concentrations from 1.0 vol. % to 3.0 vol. % (Figure 5), this activity was the lowest among the strains tested (Figure 5). However, respiration experiments were carried out at of 28 °C, a non-optimal growth temperature for this strain, which may not be relevant for assessing the biodegradation ability of psychrophilic bacteria.

Unfortunately, the complete removal of diesel fuel by selected *Rhodococcus* strains was not achieved in this work. This could be due to a short period of biodegradation (8 days) and the gradual accumulation of recalcitrant components of diesel fuel, such as isoprenoids, dominated by phytane and pristane, polyaromatic hydrocarbons, and organosulphur compounds in the growth medium [6,11,33]. From the respirometry and growth kinetics data, we assumed that a stage of active metabolism of diesel fuel was relatively rapid, but biodegradation did not stop after 8 days, when the process was no longer monitored. This active stage, similar in duration, was observed in all experimental variants and resulted, for example, in the same biodegradation percentages for the *R. ruber* IEGM 442 cells at 2.0 and 8.0 vol. % diesel fuel (see Table 4). As shown in Figure 2, the active biodegradation of 8 vol. % diesel fuel began only after 6 days, while at lower diesel fuel concentrations, the lag phase ranged from 13–24 h to 3 days, after which the cells started to metabolise diesel fuel (Figure 5 and Figure 6). It seems that rhodococci would not have enough time to degrade as much diesel fuel at 8.0 vol. % as at 2 vol. %. Thus, assuming that active metabolism occurs within 2–5 days, all available components of diesel fuel could be significantly degraded in 8 days, and similar biodegradation percentages could be achieved at all studied diesel fuel concentrations.

The GC-MS analysis (Figure 3 and Figure 4) showed a significant amount of undegraded *n-*alkanes and no pronounced changes in representative *n*-alkanes (C_15_–C_17_) during the biodegradation of 8.0 vol. % diesel fuel by *R. ruber* IEGM 442. This could be related to the preferential consumption of lower molecular weight alkanes, e.g., *n-*decane or *n-*tetradecane, which were not detected in all samples of residual diesel fuel. This finding was partially supported by respiration curves having a zigzag character (Figure 5), which suggested a sequential biodegradation of individual diesel fuel components by individual *Rhodococus* strains. The bacterial preference of certain compounds would depend on their abundance and availability. The latter depends on the mobility of hydrocarbons in NAPLs (non-aqueous phase liquids, e.g., diesel fuel in our study) and their relative solubility in water [22,24]. This is partially demonstrated in the experiments with *Rhodococcus-*biosurfactants. Their presence enhanced the biodegradation of representative alkanes, namely, they accelerated the removal of *n-*hexadecane and stimulated the degradation of *n-*pentadecane and *n-*heptadecane (Figure 4). Biosurfactants, added at double the CMC value, emulsified and dispersed NAPLs, thereby facilitating their contact with cells and the transportation of emulsified hydrocarbons through the cell wall to the membrane for further oxidation [22].

Another important finding of this study is the absence of catabolite repression in the assimilation of diesel fuel, and apparently all other hydrocarbons, by *Rhodococcus*. As revealed in co-substrate experiments, diesel fuel can be metabolised simultaneously with carbohydrates (Table 8). The best growth of *Rhodococcus* cells (2.40×10^7^ CFU·mL^−1^) was obtained with the simultaneous presence of diesel fuel and sugar in the culture medium (Table 7). The lack of catabolite repression is a useful adaptation of *Rhodococcus*, allowing the cell population to be maintained in competition with rapidly growing microbial species and to metabolise a great variety of organic compounds (as growth or co-metabolic substrates, or for the neutralization of their toxic effects). However, an increased (>2.5 g·L^−1^) sugar concentration inhibited the biodegradation of diesel fuel by two to three times (Figure 7a and Table 8), probably due to the interfering mutual influence of parallel metabolic pathways; the dominance of one of them could probably depend on the ratio between substrate concentrations. This should be taken into account when designing bioremediation processes, and the monitoring of carbohydrate concentrations would be recommended for organic-rich and hydrocarbon-contaminated sites.

## 5. Conclusions

This study provides new data on the biology of *Rhodococcus* actinomycetes, biodegraders of diesel fuel, and specific details on the microbial degradation of diesel fuel. A high resistance of *Rhodococcus* cells to diesel fuel was demonstrated. The growth of *Rhodococcus* cells can be significantly inhibited on first contact with this petroleum product, but after a period of time (e.g., 6–7 days in this study), they adapt to its toxic effect and grow in the presence of up to 32.0 vol. % diesel fuel. Although resistance is a rather specific feature of a particular strain, *R. ruber* strains are the most promising in terms of their resistance and degradation activity. According to our results, the inhibitory concentrations of diesel fuel in the RS medium against representatives of *R. ruber* are 8.0–16.0 vol. %, and some *R. ruber* strains (e.g., IEGM 442) are able to degrade diesel fuel at these high concentrations. Another important point for bioremediation is the dependence of strain degradation activities on temperature. The strain *Rhodococcus* sp. IEGM 1276 was most active in degrading diesel fuel at 4 °C. There was no evidence of catabolite repression in the biodegradation of diesel fuel by *Rhodococcus*, which occurred in the presence of other carbon sources, such as granular sugar and yeast extract. This feature gives *Rhodococcus* a competitive advantage and they grow better in media containing both diesel fuel and sugar. However, the high concentration of additional carbon sources has a negative effect on the efficiency of diesel fuel biodegradation. In particular, sugar at concentrations > 2.5 g·L^−1^ inhibits the biodegradation process. The recommended growth conditions are 1.3 g·L^−1^ of granular sugar and 0.25 g·L^−1^ of yeast extract, which allowed for the biodegradation of 71–88% diesel fuel.

The strains selected in this work on the basis of their resistance and degradation activities are *R. ruber* IEGM 231, IEGM 442, and *Rhodococccus* sp. IEGM 1276. They can be recommended for the bioremediation of diesel fuel-contaminated sites. *R. ruber* IEGM 442 was best suited for heavy contamination (up to 8.0 vol. % diesel fuel) at a standard temperature (28 °C) and *Rhodococcus* sp. IEGM 1276 for light contamination (not more than 2.0 vol. % diesel fuel) at a low temperature (4 °C). *R. ruber* IEGM 231 is suitable for diesel fuel contamination up to 3.0 vol. % at 28 °C. However, it has the highest respiratory rates under these conditions, which is its advantage over *R. ruber* IEGM 442. Further identification of *Rhodococcus* sp. IEGM 1276 is required for bioremediation applications. Its draft genome has been sequenced and can be provisionally assigned to *Gordonia amicalis* (DDBJ/ENA/GenBank acc. no JAPWIL010000001–JAPWIL010000081, accessed 15 November 2024). We follow the polyphasic taxonomy approach and intend to harmonise phenotypic and genotypic data before the final strain identification, which is the subject of future research.

The use of externally added *Rhodococcus-*biosurfactants at a concentration of 1.4 g·L^−1^ has proven to be efficient for hydrocarbon biodegradation. Although no statistically significant effects of the biosurfactants on the total removal of diesel fuel were determined, the biodegradation of individual *n-*alkanes (*n-*pentadecane, *n-*hexadecane, and *n-*heptadecane) was stimulated, which may accelerate the bioremediation process. No additional contamination with *n-*hexadecane was detected when biosurfactants were added, although they were produced by the *R. ruber* IEGM 231 cells grown with 3 vol. % *n-*hexadecane (see Section 2.1). The total removal of more than 71% of diesel fuel in 8 days can be expected when using selected strains and biosurfactants under the optimised growth conditions.

This work requires further investigation, focusing on field trials, the development of consortia, and the construction of stable biocatalysts. Selected *Rhodococcus* strains appear to be environmentally compatible, with no possible adverse effects on indigenous microorganisms. *Rhodococcus* actinomycetes are ubiquitous in biotopes throughout the world, are dominant in hydrocarbon-contaminated ecosystems, are tolerant to fluctuations in abiotic factors, and their abundance returns to background levels after the end of the intensive phase of bioremediation, without harmful effects on microbial communities [23,27,50].

## Figures and Tables

**Table 1:** *Rhodococcus* strains used in this study.

Strain	Isolation Source
*R. erythropolis* IEGM 251	Snow, Yakutia, Saha Republic, Russia
*R. erythropolis* IEGM 275	Oil-polluted soil, oil-extracting enterprise, Perm region, Russia
*R. erythropolis* IEGM 587	*Tussilago farfara* rhizosphere, techogenically polluted soils, Gubakha, Perm region, Russia
*R. erythropolis* IEGM 1189	Water, Tyumen region, Russia
*R. jostii* IEGM 60	Oil-polluted soil, oilfield, Ukraine
*R. opacus* IEGM 249	Soil, lavsan (polyester fibre) production, Belarus
*R. opacus* IEGM 717	Soil
*R. opacus* IEGM 1157	Plantago rhizosphere, soil, former landfill area, Perm, Perm region, Russia
*R. qingshengii* IEGM 267	Oil-polluted soil, oil-extracting enterprise, Perm region, Russia
*R. qingshengii* IEGM 1359	Bottom sediment from lake systems, Li Smita Island, Franz Josef Land, Arkhangel’sk region, Russia
*R. rhodochrous* IEGM 639	Snow, oilfield, Perm region, Russia
*R. rhodochrous* IEGM 1137	Oil-polluted soil, Solikamsk, Perm region, Russia
*R. rhodochrous* IEGM 1138	Oil-polluted soil, Solikamsk, Perm region, Russia
*R. ruber* IEGM 231	Water, spring, Olkhovski oil-extracting enterprise, Perm region, Russia
*R. ruber* IEGM 234	Snow, Polazna oil-extracting enterprise, Perm region, Russia
*R. ruber* IEGM 442	Snow, oilfield, Perm region, Russia
*R. ruber* IEGM 1263	Oil-polluted waste, Sosnogorsk, Komi Republic, Russia
*Rhodococcus* sp. IEGM 1276	Oil slime, Udmurt Republic, Russia

**Table 2:** Levels for carbon sources in multifactor analysis.

Level	Diesel Fuel Concentration, vol. %	Sugar Concentration, g·L^−1^	Yeast Extract Concentration, g·L^−1^
+1	2.0	5.0	1.00
0	1.0	2.5	0.50
−1	0.5	1.3	0.25

**Table 3:** Toxicity of diesel fuel to *Rhodococcus* cells.

Strain	Inhibitory Concentration of Diesel Fuel, vol. %			
	LB		RS	
	3 Days *	7 Days	3 Days	7 Days
*R. erythropolis* IEGM 251	8.0	8.0	8.0	8.0
*R. erythropolis* IEGM 1189	8.0	16.0	0.5	0.5
*R. jostii* IEGM 60	0.5	32.0	0.5	0.5
*R. opacus* IEGM 249	4.0	32.0	4.0	4.0
*R. opacus* IEGM 717	8.0	8.0	0.5	0.5
*R. opacus* IEGM 1157	8.0	16.0	0.5	1.0
*R. qinshengii* IEGM 267	16.0	16.0	4.0	4.0
*R. qinshengii* IEGM 1359	4.0	32.0	0.5	0.5
*R. rhodochrous* IEGM 639	32.0	32.0	4.0	16.0
*R. rhodochrous* IEGM 1137	16.0	64.0	4.0	4.0
*R. rhodochrous* IEGM 1138	8.0	8.0	0.5	0.5
*R. ruber* IEGM 231	64.0	64.0	8.0	8.0
*R. ruber* IEGM 234	0.5	4.0	8.0	8.0
*R. ruber* IEGM 442	32.0	32.0	16.0	16.0
*R. ruber* IEGM 1263	32.0	32.0	8.0	8.0
*Rhodococcus* sp. IEGM 1276	64.0	64.0	4.0	4.0

* MIC—minimal inhibitory concentration.

**Table 4:** Total removal and microbial degradation of diesel fuel using *Rhodococcus* cells after 8 days.

Strain	Concentration of Diesel Fuel, vol. %	Temperature			
		4 °C		28 °C	
		Total Removal, %	Microbial Degradation, %	Total Removal, %	Microbial Degradation, %
*R. ruber* IEGM 231	2.0	24 *	10	86 *	44
*R. ruber* IEGM 442	2.0	29 *	15	84 *	42
*R. ruber* IEGM 442	8.0	Not analysed	Not analysed	82 *	40
*Rhodococcus* sp. IEGM 1276	2.0	73 *	59 **	75 *	33 **
Abiotic control (no cells)	2.0	14	Not applicable	43	Not applicable
Abiotic control (no cells)	8.0	Not analysed	Not analysed	42	Not applicable

* Statistically significant from abiotic control at *p* < 0.05. ** Statistically significant from other strains at *p* < 0.05.

**Table 5:** Kinetic parameters of *Rhodococcus* sp. IEGM 1276 growth in the presence of diesel fuel at 4 °C.

Diesel Fuel Concentration, vol. %	Y	µ, h^−1^	X, g·L^−1^
2.0	0.069	0.138	0.070
3.0	0.005	0.312	0.039

**Table 6:** Metabolism of sugar by *Rhodococcus* cells in the presence of diesel fuel at 28 °C in 7 days.

Strain	Diesel Fuel Concentration, vol. %	Sugar Concentration, g·L^−1^	Growth	Removal of Sugar, %
*R. erythropolis* IEGM 587	2.0	5.0	++	56
	2.0	10.0	+++	73 *
*R. erythropolis* IEGM 275	2.0	5.0	++	33
	2.0	10.0	+++	61 *

“++/+++”—Relative intensity of the cell growth from weak to strong. * Statistically significant from the sugar concentration of 5 g·L^−1^ at *p* < 0.05.

**Table 7:** Growth of *R. erythropolis* IEGM 587 cells in RS with different combinations of growth substrates.

Sugar Concentration, g·L^−1^	Diesel Fuel Concentration, vol. %	Yeast Extract Concentration, g·L^−1^	Number of Cells, ×10^7^ CFU·mL^−1^	Removal of Sugar, %
2.5	0.0	0.25	0.12 ± 0.01 *	64
0.0	2.0	0.25	1.65 ± 0.11 *	Not applicable
2.5	2.0	0.00	2.40 ± 0.16 *	55

Time of cultivation was 7 days. Temperature of cultivation was 28 °C. * Statistically significant from other trials at *p* < 0.05.

**Table 8:** Growth and efficiency of growth substate utilisation by *R. erythropolis* IEGM 587 cells at different concentrations of sugar, diesel fuel, and yeast extract used simultaneously.

Concentration of Sugar/YE *, g·L^−1^	Biodegradation of Diesel Fuel, %			Number of Cells, ×10^7^ CFU·ml^−1^			Removal of Sugar, %		
	Diesel Fuel Concentration, vol. %								
	0.5	1.0	2.0	0.5	1.0	2.0	0.5	1.0	2.0
2.5/0.25	86	79	82	3.40 ± 0.27	1.85 ± 0.33	2.50 ± 0.25	79	64	46
5.0/0.25	54	28	36	3.85 ± 0.22	3.55 ± 0.03	3.95 ± 0.19	83	61	73
2.5/0.50	87	69	64	3.85 ± 0.11	4.25 ± 0.27	3.45 ± 0.21	77	20	58
5.0/0.50	27	16	21	4.45 ± 0.16	3.15 ± 0.06	3.25 ± 0.07	70	39	49
2.5/1.00	91	80	57	3.20 ± 0.39	3.40 ± 0.09	3.95 ± 0.04	32	46	48
5.0/1.00	53	65	48	4.60 ± 0.17	4.20 ± 0.11	3.80 ± 0.14	46	54	62
1.3/0.25	84	82	88	1.90 ± 0.07	2.05 ± 0.08	0.65 ± 0.08	70	36	77
1.3/0.50	73	87	88	0.45 ± 0.02	0.80 ± 0.11	0.50 ± 0.09	58	27	60
1.3/1.00	71	76	86	1.95 ± 0.04	1.80 ± 0.13	1.35 ± 0.06	68	49	66

Time of cultivation was 7 days. Temperature of cultivation was 28 °C. * YE—yeast extract.

## Data Availability

The raw data supporting the conclusions of this article will be made available by the authors on request.

## Supplementary Materials

The following supporting information can be downloaded at: https://www.mdpi.com/article/10.3390/microorganisms12122605/s1, Table S1: “Indexing carbon source combinations for multifactor analysis”; Figure S1: “Example of toxicity test results. INT staining of *Rhodococcus ruber* IEGM 442 cells in the presence of diesel fuel after incubation at 600 min^−1^, 28 °C for 3 days. Upper wells (rows A–D) are not stained and left for the next 4 days to determine bacteriostatic/bactericidal effect of diesel fuel at inhibitory concentrations. Abiotic control: LB or RS with 0.5 vol. % of diesel fuel without cells. Biotic control: inoculated LB or RS with 3 vol. % *n-*hexadecane”.
