# Supplementary material for: Resistant Rhodococcus for Biodegradation of Diesel Fuel at High Concentration and Low Temperature
Source: Microorganisms. 2024 Dec 17;12(12):2605. doi: 10.3390/microorganisms12122605 (PMC11676119; doi:10.3390/microorganisms12122605)
Supplement: Supplementary file 1 [file microorganisms-12-02605-s001.zip › microorganisms-3356157-supplementary.pdf]

Table S1. Indexing carbon source combinations for multifactor analysis.

Index	Diesel fuel level	Sugar level	Yeast extract level	Index	Diesel fuel level	Sugar level	Yeast extract level
1	-1	0	-1	15	0	+1	0
2	0	0	-1	16	0	+1	+1
3	+1	0	-1	17	+1	+1	0
4	-1	0	+1	18	+1	+1	+1
5	-1	0	0	19	-1	-1	-1
6	0	0	0	20	-1	-1	0
7	0	0	+1	21	-1	-1	+1
8	+1	0	0	22	0	-1	-1
9	+1	0	+1	23	0	-1	0
10	-1	+1	-1	24	0	-1	+1
11	0	+1	-1	25	+1	-1	-1
12	+1	+1	-1	26	+1	-1	0
13	-1	+1	0	27	+1	-1	+1
14	-1	+1	+1				

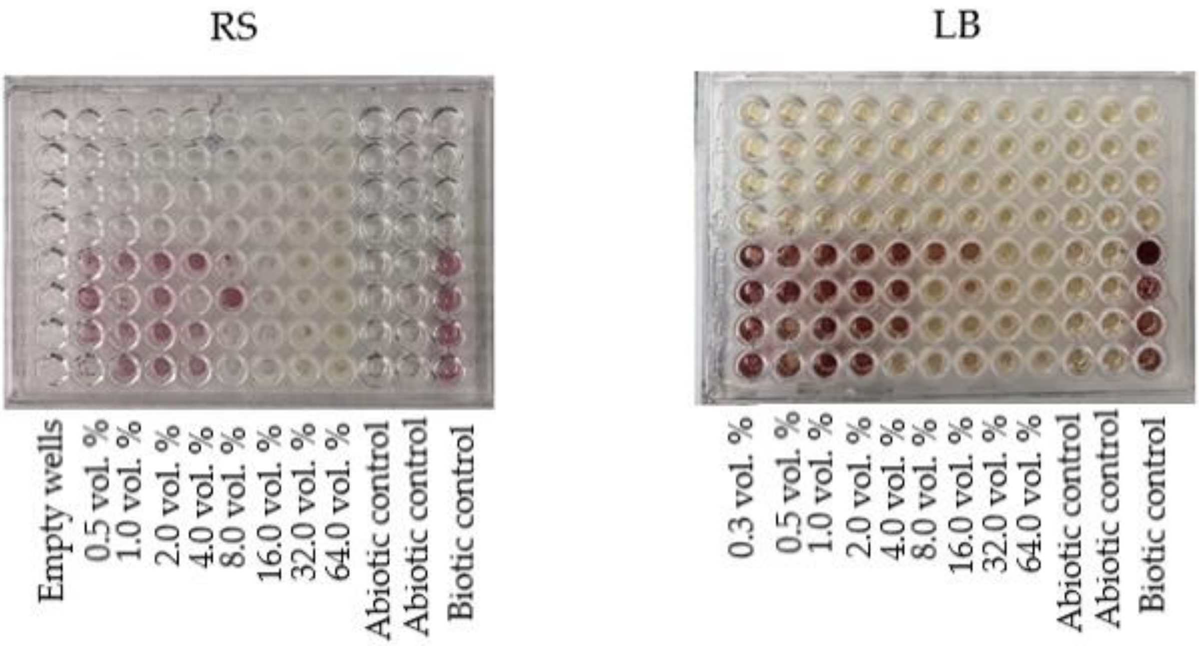

Figure S1. Example of toxicity test results. INT staining of *Rhodococcus ruber* IEGM 442 cells in the presence of diesel fuel after incubation at 600 min^{-1}, 28°C for 3 days. Upper wells (rows A–D) are not stained and left for the next 4 days to determine bacteriostatic / bactericidal effect of diesel fuel at inhibitory concentrations. Abiotic control: LB or RS with 0.5 vol. % of diesel fuel without cells. Biotic control: inoculated LB or RS with 3 vol. % *n*-hexadecane.
